# *Trichoderma*-Plant Root Colonization: Escaping Early Plant Defense Responses and Activation of the Antioxidant Machinery for Saline Stress Tolerance

**DOI:** 10.1371/journal.ppat.1003221

**Published:** 2013-03-14

**Authors:** Yariv Brotman, Udi Landau, Álvaro Cuadros-Inostroza, Tohge Takayuki, Alisdair R. Fernie, Ilan Chet, Ada Viterbo, Lothar Willmitzer

**Affiliations:** 1 Max Planck Institute of Molecular Plant Physiology, Am Mühlenberg 1, Potsdam-Golm, Germany; 2 Department of Plant Pathology and Microbiology, Robert H. Smith Faculty of Agriculture, Food and Environment, The Hebrew University of Jerusalem, Rehovot, Israel; University of Melbourne, Australia

## Abstract

*Trichoderma* spp. are versatile opportunistic plant symbionts which can colonize the apoplast of plant roots. Microarrays analysis of *Arabidopsis thaliana* roots inoculated with *Trichoderma asperelloides* T203, coupled with qPCR analysis of 137 stress responsive genes and transcription factors, revealed wide gene transcript reprogramming, proceeded by a transient repression of the plant immune responses supposedly to allow root colonization. Enhancement in the expression of *WRKY18* and *WRKY40*, which stimulate JA-signaling via suppression of JAZ repressors and negatively regulate the expression of the defense genes *FMO1, PAD3* and *CYP71A13*, was detected in *Arabidopsis* roots upon *Trichoderma* colonization. Reduced root colonization was observed in the *wrky18*/*wrky40* double mutant line, while partial phenotypic complementation was achieved by over-expressing *WRKY40* in the *wrky18 wrky40* background. On the other hand increased colonization rate was found in roots of the FMO1 knockout mutant. *Trichoderma* spp. stimulate plant growth and resistance to a wide range of adverse environmental conditions. *Arabidopsis* and cucumber (*Cucumis sativus* L.) plants treated with *Trichoderma* prior to salt stress imposition show significantly improved seed germination. In addition, *Trichoderma* treatment affects the expression of several genes related to osmo-protection and general oxidative stress in roots of both plants. The MDAR gene coding for monodehydroascorbate reductase is significantly up-regulated and, accordingly, the pool of reduced ascorbic acid was found to be increased in *Trichoderma* treated plants. 1-Aminocyclopropane-1-carboxylate (ACC)-deaminase silenced *Trichoderma* mutants were less effective in providing tolerance to salt stress, suggesting that *Trichoderma*, similarly to ACC deaminase producing bacteria, can ameliorate plant growth under conditions of abiotic stress, by lowering ameliorating increases in ethylene levels as well as promoting an elevated antioxidative capacity.

## Author Summary

*Trichoderma* fungi have been developed as biocontrol agents and are applied to protect and improve crop yields. Colonization of plant roots by *Trichoderma* can protect plants against diseases and environmental stresses such as salinity and drought, and an improve plant growth and development. To better understand the mechanism underlining the plant-*Trichoderma* interaction we followed changes in global gene expression in colonized *Arabidopsis* roots. We associate the known gene biological function to the processes of root colonization and abiotic stress tolerance mediated by *Trichoderma*. Using *Arabidopsis* mutant lines we show the function of a subset of those genes in root colonization. We show that *wrky18* and *wrky40* transcription factors activate and suppress the expression of different genes in order to allow successful root colonization. We also combine the gene expression data together with the measurement of ascorbic acid level to demonstrate that salt stress tolerance offered by *Trichoderma* is dependent on activation of the plant antioxidant defense machinery. Using *Trichoderma* lines mutated in the ACC deaminase gene, we demonstrate that reduction of ethylene levels is also essential in achieving salt tolerance. This study represents an important step forward in understanding the nature of the non-pathogenic plant *Trichoderma* interaction, and may contribute to the efforts to improve *Trichoderma* biocontrol abilities.

## Introduction

*Trichoderma* spp. are endophytic plant opportunistic symbionts widely used as biofertilizers and biocontrol agents for plant diseases [1], [2]. Inhibition of plant disease severity is achieved through direct antagonism and mycoparasitism of the plant pathogens in the soil or on plant roots [3]. Although, some *Trichoderma* rhizosphere-competent strains can also colonize root intercellular spaces [4] and have direct effects on plants including increased growth and nutrient uptake, increased fertilizer efficiency utilization, percentage and rate of seed germination, and induced systemic resistance (ISR) to diseases [5]. Abiotic stresses frequently limit growth and productivity of major crop species, reducing yields to less than half of that possible under ideal growing conditions [6]. *Trichoderma* abilities to alleviate abiotic stresses are known, although specific knowledge of mechanisms controlling multiple plant stress factors is still lacking [7]. reported that *Trichoderma harzianum* T22 treated seeds germinated faster and more uniformly than untreated seeds whether the stress applied was osmotic, salt, or suboptimal temperatures. Application of the antioxidant glutathione, or application of T22, resulted in a similar positive effect. Recently [8], demonstrated that T22 improves tolerance to water deficit of tomato seedlings by enhancing the antioxidant defense mechanism and higher activity of ascorbate and glutathione-recycling enzymes. This finding thus supports the model that *T. harzianum* strain T22 increases seedling vigor and ameliorates stress by inducing physiological protection in plants against oxidative damage.

These data are comparable to the effects induced in plants by *Piriformospora indica*, a plant-root-colonizing basidiomycete fungus which can provide strong growth-promoting activity during its symbiosis with a broad spectrum of plants [9], and can induce resistance to fungal diseases and tolerance to salt stress that have been associated with an elevated antioxidative capacity [10]–[12]. Another mechanism for protecting plants against environmental stress has been demonstrated in plant growth promoting bacteria (PGPR), encoding the enzyme 1-aminocyclopropane-1-carboxylate deaminase (ACCD) (E.C. 4.1.99.4) that cleaves (ACC), the immediate precursor of ethylene [13]. A role for ACCD in the plant root growth-promotion effect by *Trichoderma* has been demonstrated by RNAi silencing of the ACCD gene in *T. asperelloides* showing decreased ability of the mutants to promote root elongation of canola seedling [14].

Within the last years, a few studies have reported on global transcriptome and proteome changes in plants colonized with *Trichoderma*. These studies led to the identification of genes and proteins that are likely involved in the beneficial interaction between *Trichoderma* and different plants species in root [15]–[17] and in leaf after the onset of induced systemic resistance (ISR) [18], [19]. Recently, using microarray analysis [20] showed global gene expression changes in aerial part of *Arabidopsis* 24 hours after roots inoculation with *Trichoderma harzianum* T34. Most of the detected *Arabidopsis* defense-related genes, mediated by jasmonic acid (JA) and salicylic acid (SA), appear to be down-regulated by *T. harzianum* T34. Among these, genes involved in systemic acquired resistance (SAR) responses such as *FMO1* (flavin monooxygenase 1) and *PR-1* (pathogenesis-related 1), a marker for the SA dependent SAR response [21] are down-regulated. These findings suggest that *Trichoderma*, like the plant beneficial fungus *Piriformospora indica*, has to cope with plant defense responses during the initial stages of the interaction by broad-spectrum suppression of innate immunity, to allow colonization of *Arabidopsis* roots [22].

Induction of defense responses is typically associated with the coordinated transcriptional modulation of large numbers of genes [23]. WRKY DNA binding transcription factors have important roles in the regulation of genes associated with plant defense responses [24]. WRKY transcription factors function as positive and negative regulators of plant defense response has been demonstrated using *Arabidopsis* mutants [25], [26]. A few studies also show the additive function of WRKY TF, demonstrating the web-like nature of the WRKY TF family [24]. For example, *WRKY38* and *WRKY62* have been shown to be negative regulators of plant basal defense in *Arabidopsis* [27]. Another example of additive mode of action of WRKY TF is the concomitantly action of WRKY18, WRKY40 and WRKY60 genes in *Arabidopsis* [28]. showed that *WRKY18*, *WRKY40* and *WRKY60* form homo- and hetero-complexes. When *Arabidopsis* were simultaneously mutated in *WRKY18* and *WRKY40* they gain better resistant to the biotrophic fungus *Golovinomyces orontii* [29], and to the hemibiotrophic bacterium *Pseudomonas syringe* [28]. On the other hand, *WRKY18* and *WRKY40* double mutants show higher susceptibility to the necrotrophic fungus *Botrytis cinerea* [28]. [30] showed that *WRKY18* and *WRKY40* negatively modulate the expression of positive regulators of defense such as *CYP71A13*, *EDS1* and *PAD4*, but positively modulate the expression of some key JA-signaling genes by partly suppressing the expression of JAZ repressors. Hence, those TF are in the point of convergence between different defense signaling pathways.

In this study we follow the global gene expression in *Arabidopsis* roots colonized by *Trichoderma* and we show that 24 hours after the onset of colonization there are profound changes in plant transcripts associated to resistance to both biotic and abiotic stresses. Two main aspects of plant-*Trichoderma* interaction are highlighted by our analysis: plant gene modulation during root colonization by the endophyte and, tolerance to abiotic stresses conferred to the plant. Thus, by conducting further experiments using *Arabidopsis* mutant lines, cucumber and *Arabidopsis* plants exposed to salt stress with or without *Trichoderma* pre-inoculation, with *Trichoderma* wild-type or a ACC-deaminase gene mutant line, we provide evidence that: (i) during root colonization, *Trichoderma* manipulates WRKY18 and WRKY40 transcription factors activities to modulate the expression of the JAZ repressor genes and defense response genes, such as *FMO1* and *CYP71A13* to its advantage in the same way of biotrophic and hemibiotrophic plant pathogens (ii) *Trichoderma* fungi can ameliorate plant growth under abiotic stressful conditions by lowering deleterious elevated ethylene levels accompanied by an elevated antioxidative capacity.

## Results

### Plant roots colonized by *Trichoderma* display pronounced changes in expression of transcripts involved in stress responses

**Figure 1:**
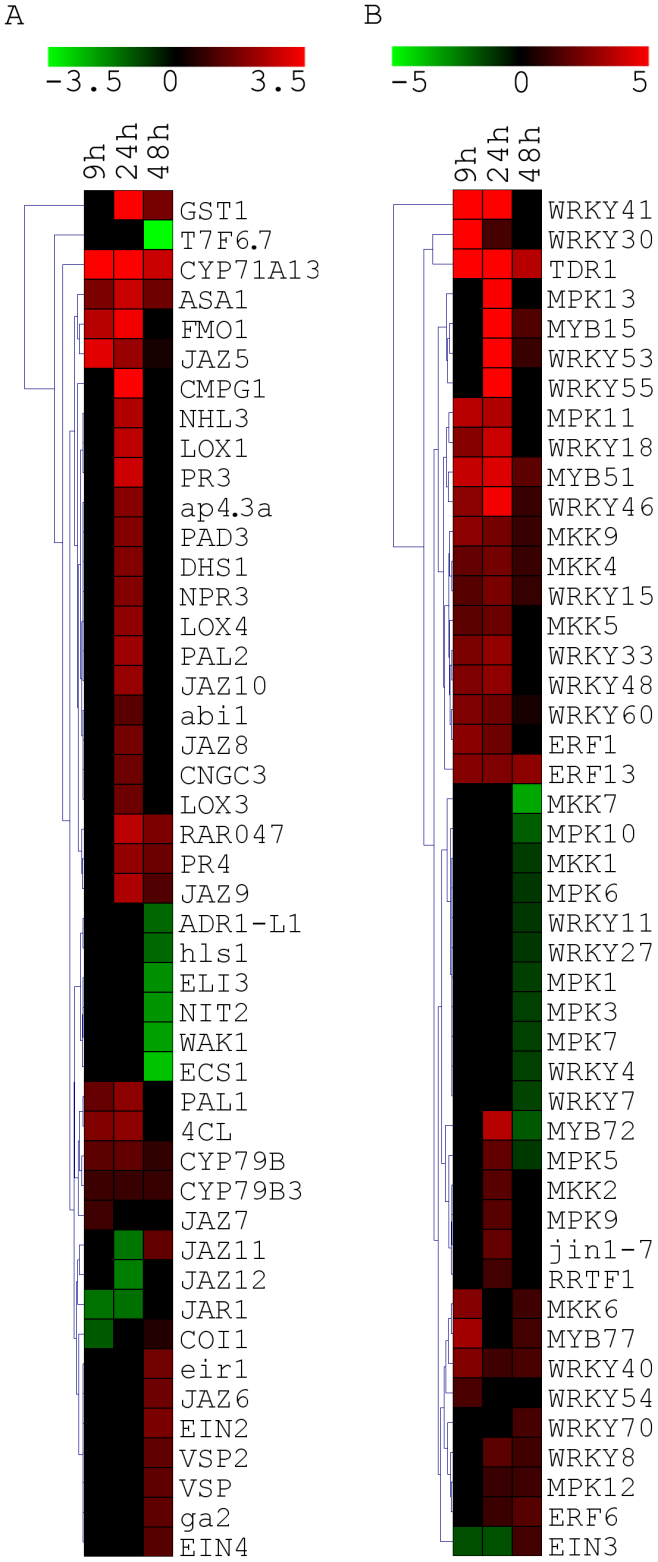
Hierarchical clustering representation of genes expression quantified by qPCR in *Arabidopsis* roots. Euclidean distance and average linkage were used to construct the clustering of biotic and abiotic stress responsive genes (A) and transcription factors (B). Roots were collected at 9, 24 and 48 hpi by *T. asperelloides* T203. Each cell represents the fold expression average of six independent biological repetitions of each time point, and is relative to control collected in each one of the time points. Black: no significant difference (*P*>0.05), red-up regulation, green-down regulation significantly different from control (*P*<0.05).

**Figure 2:**
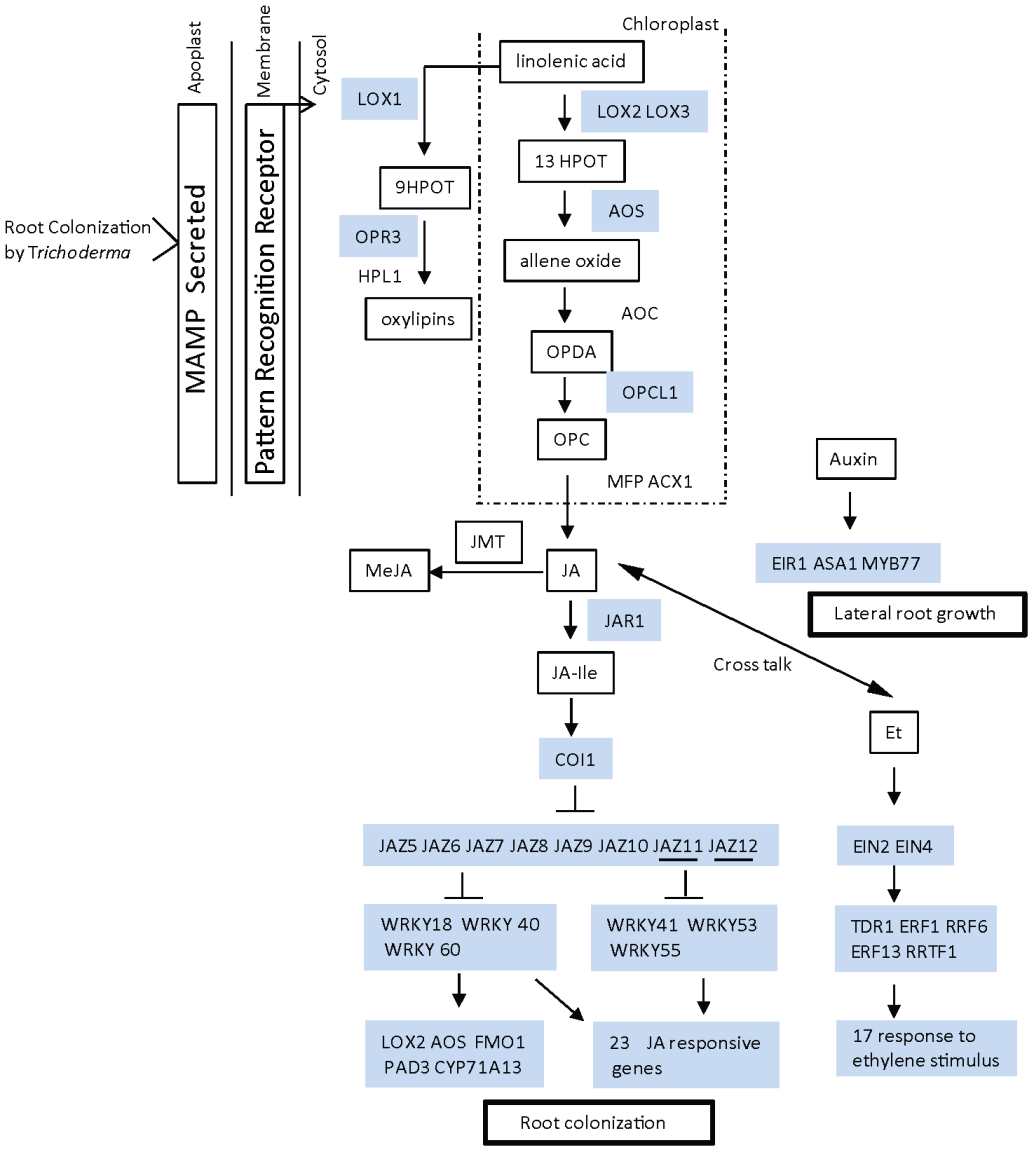
Overview of modulation of expression of phyto-hormone biosynthesis and responsive genes in *Arabidopsis* roots during *Trichoderma* colonization. In the early steps of the interaction, *Trichoderma* secreted MAMPs that triggered activation of signal transduction which modulated the expression of several genes. Among them, genes that have been previously shown to have a role in biosynthesis and response to JA, ethylene (Et; significant enrichment, FDR: *P*<0.01) and auxin (non-significant enrichment). Based on previously published data, we propose two main biological roles for the activation of those phyto-hormone related pathways in the plant-*Trichoderma* interaction: (i) colonization regulated by JA and ethylene, and (ii) lateral root formation regulated by JA and ethylene. Intermediate metabolites in the biosynthesis pathways are indicated by bold frame. Genes mediating the biosynthesis process are indicated without frame or marked in blue in case they show a significant (P<0.05) change in expression level upon *Trichoderma* colonization. Down-regulated genes are underlined. The model is based on the following published studies [28], [30], [39]–[41], [50], [76]–[78] and the oxylipins pathway (based on KEGG pathways). Gene names are in conformity with TAIR annotation.

To monitor global changes in genes expression following colonization of *Arabidopsis* roots by *Trichoderma* we performed microarray analysis 24 hours after the application of *Trichoderma* to the roots. Results indicate that *Trichoderma* root colonization affects the root transcriptome, with 249 probe sets showing an increased expression (>2 fold, p<0.0001) and 29 probe sets indicating a decreased expression (>2 fold, p<0.0001) after data filtering (File S1). Those 278 genes, showing significant change in expression, were subjected to Singular Enrichment Analysis (SEA) in order to identify enriched Gene Ontology (GO) terms [31]. Enriched functional categories were sorted according to statistically significant enrichment together with the gene identifier observed for each functional category (File S2). A substantial (False Discovery Rate (FDR)<0.01), enrichment in genes correlated with response to biotic and abiotic stress (15% and 14% respectively, Table 1), response to different stimuli such as carbohydrate and chitin, as well as genes involved in hormone biosynthesis and response to hormone signaling, was detected. Interestingly, 7% of the total up-regulated genes are related (FDR<0.01) to the oxylipin biosynthetic process (*OPR3*, *AOS*, *OPCL1*, *LOX2, LOX3* and *LOX4* genes), leading to JA biosynthesis (File S2, Table 1). A summary of the genes up-regulated in the microarray experiments with known role in plant hormone biosynthesis process and responses to hormone stimulation is presented in File S3. Among the down-regulated transcripts, SEA analysis did not reveal any significant enrichment in any class of GO-terms. Nevertheless noteworthy, were four members of the plant cytochrome P450 monooxygenases (CYP) family which were down-regulated. In *Arabidopsis*, CYP genes family includes 245 genes and constitutes up to 1% of the protein coding genes [32]. Co-expression analysis and data base search using STRING version 9.0 [33] and co-regulatory networks (ATTED-II; http://atted.jp/) did not, however, reveal any known correlation between those genes.Expression analysis was further performed using a profiling platform covering 137 biotic and abiotic stress responsive genes and transcription factors (List of genes and primers can be found in file S4). Transcript modulation by *Trichoderma* was followed by qPCR 9, 24 and 48 hours post inoculation (hpi). Heatmap representations (Figure 1A and 1B) of significantly affected genes (p<0.05) reveal transient transcript activation during the first hours of interaction. Noteworthy, genes related to ethylene/JA regulation (*WRKY41*, *WRKY53*, *WRKY55*, *ERF1*, *ERF6*, *ERF13* and *RRTF1*) (Figure 1B), JA signaling (*WRKY18*, *WRKY40*, *WRKY33*, *JIN1*-7 and *TDR1*), root development and auxin modulation (*MYB77*), and secondary metabolites synthesis (*MYB51*). *WRKY33* and *MYB51* are also key elements of salt/osmotic stress regulation. Of note among other genes, mostly with function in general related defense responses and biotic and abiotic signaling process (Figure 1A), are genes related to JA biosynthesis, *LOX1*, *LOX3* and *LOX4*,and repressors of JA responses, (JAZ gene family) that show increased (*JAZ5*, *JAZ6*, *JAZ7*, *JAZ8*, *JAZ9*, and *JAZ10*) or decreased (*JAZ11* and *JAZ12*) expression. Moreover, we observed an increase in expression of JA-responsive genes, such as *VSP*, *VSP2* and *PAD3*. Increased expression of some of those genes such as *WRKY18*, *JAZ8*, *JAZ10, LOX3* and *LOX3* was also detected in the microarray analysis (File S1). Other *Trichoderma* modulated genes belong to the ethylene-signaling pathway, such as *EIN2* and *EIN4* which are positive regulators of ethylene responses. Auxin related genes include, *ASA1* that function in jasmonate mediated regulation of auxin biosynthesis and lateral root formation and *eir1* involved in auxin transport (Figures 1A). As a large protion of the *Trichoderma* responsive genes correspond to processes directly associated with JA, Ethylene and Auxins metabolism and response, we zoom in on those differentially expressed genes to obtain an overview of the changes and their possible biological role in more detail (Figure 2). Of note, increased expression was also observed in genes of the phenylpropanoid pathway, phenylalanine ammonia lyase (*PAL1* and *PAL2*) a key enzyme of the first step of the pathway and *4CL*, which is involved in the last step of the pathway. Moreover, substantially large numbers of genes that take part in different defense response processes like *PDF1.2, PR-2* and *PR-4* are up-regulated (Figures 1A and 1B, File S1). The expression of 22 genes that show increase expression in the microarray analysis are in agreement with the qPCR results, thus validating the microarray analysis (File S5). Mapman software [34] analysis further highlight the biological processes affected during *Trichoderma* colonization (Figure S1).

**Table 1:** Selected enriched functional categories

Term	% from the total number	Gene No.	FDR
response to stimulus	31.7	82	3.90E-12
response to abiotic and biotic stress	20.1	52	9.00E-10
response to organic substance	12.0	31	1.50E-06
response to other organism	7.7	20	1.30E-06
response to external stimulus	6.2	16	4.30E-06
response to carbohydrate stimulus	5.8	15	2.50E-08
response to chitin	5.4	14	9.00E-10
response to fungus	5.0	13	1.80E-08
jasmonic acid	6.9	18	2.50E-06
monocarboxylic acid metabolic process	3.8	10	0.016
response to oxidative stress	3.1	8	0.049

Enriched functional categories analyses were performed with the genes that show significant (*P*<0.05) up-regulation in the microarray hybridization (File S1). Class Identifiers with substantial, low False Discovery Rate (FDR<0.01), and with putative role in the beneficial interaction between *Trichoderma* and *Arabidopsis* are shown. Induction for the number of genes in each category is presented. Complete list of enriched functional categories is provided in File S2.

### Root colonization by *T. asperelloides* is reduced in *WRKY18* and *WRKY40* double mutant knockout line

**Figure 3:**
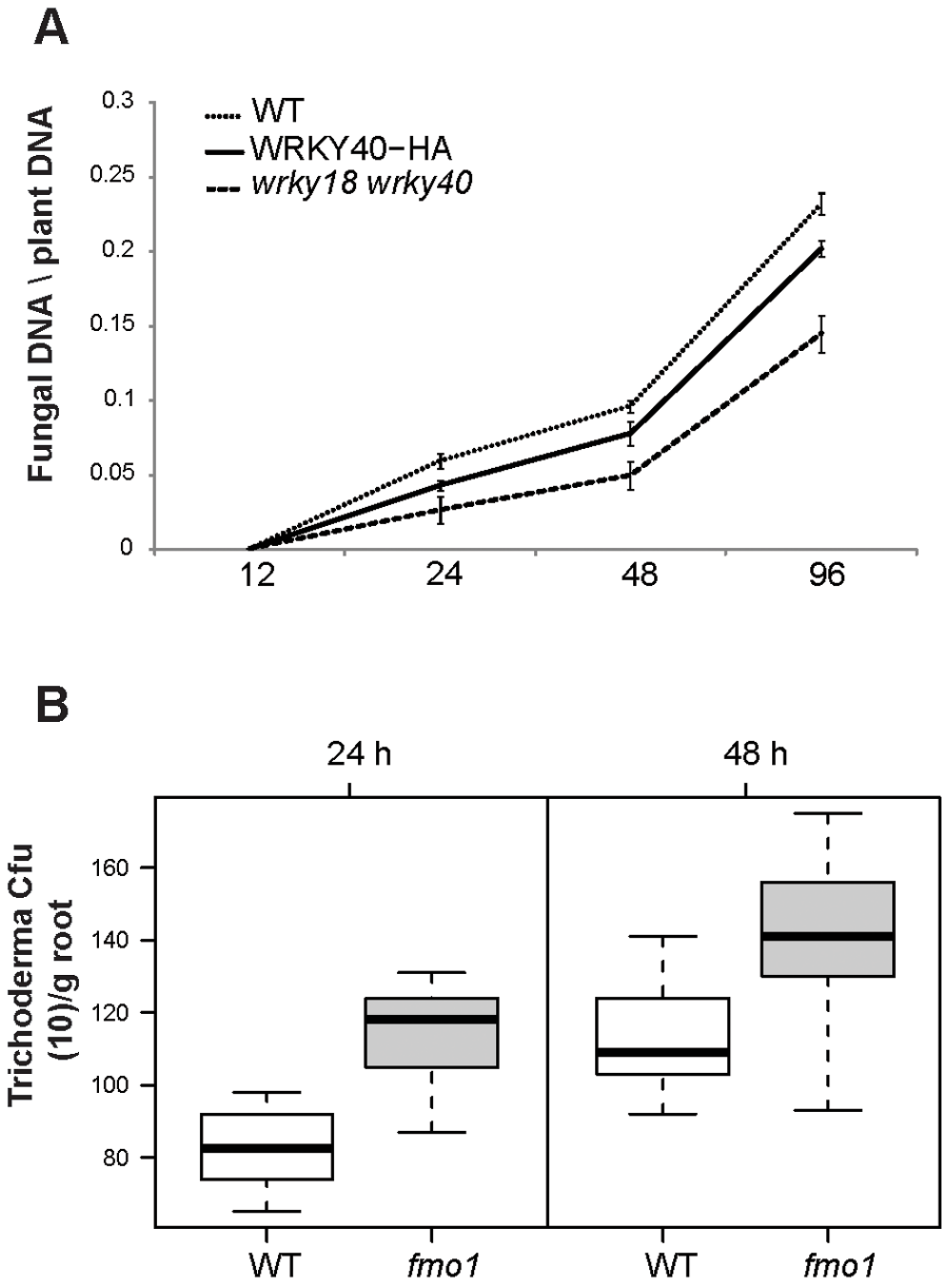
Root colonization of different *Arabidopsis* genotypes by *T. asperelloides*. Root colonization rate was quantified at different points as described in Materials and Methods section by qPCR (A) or fungal colonies count (CFU; B). The genotypes, WT (Col-0), *wrky18*/*wrky40*, WRKY40-HA complemented *wrky18*/*wrky40* and *fmo1* lines were assayed. Eight replicates were tested in each experiment, with 3 plants per treatments and the results are the average of three independent experiments. hpi: hours post-inoculation. *: = in each of the time points each of the line is significantly different from the other (*P*<0.001; *t* test).

**Figure 4:**
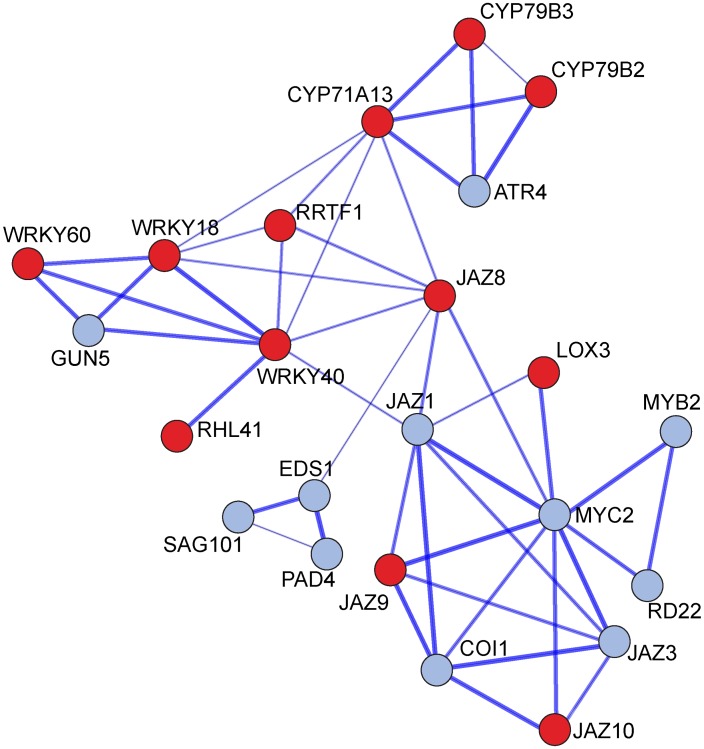
JAZ8 network based on co-expression analysis and data base search. STRING version 9.0 was used to query *JAZ8* (*AT1G30135*) gene. In the resulting network, the expression of each of the gene was monitored by qPCR 24 hpi by *T. asperelloides*. Red- gene that show significant increased expression (*P*<0.05; *t* test), grey- non significant change (*P*>0.05; *t* test).

Our expression analyses using both microarray and qPCR revealed significant up-regulation of *WRKY18*, *WRKY40* and *WRKY60* transcription factors (TF) as early as 9 hours from the onset of the interaction. Those TF genes encoding regulators of the JA signaling networks enable plants to coordinate and fine-tune responses to different pathogens [28]–[30]. To investigate whether *WRKY18* and *WRKY40* genes have a role in root colonization, we evaluated *Trichoderma* root colonization in *wrky18*/*wrky40* double knockout line and compared with WT Col-0 plants. Total DNA was extracted from roots at 12, 24, 48 and 96 hpi and the amount of *Trichoderma* fungal DNA was quantified using *Trichoderma* specific primers and normalized to plant reference genes [35]. *wrky18*/*wrky40* line exhibited reduced root colonization ability (*P*<0.05, Figure 3A). To determine if one gene was functionally sufficient to restore WT level of colonization, and to define the contribution of *WRKY40* in this process, we evaluated root colonization in a line were the *WRKY40* was over-expressed in the *wrky18*/*wrky40* background mutant [30]. *Trichoderma* colonization ability was clearly enhanced compared with the double mutant (Figure 3A), but colonization did not reach WT levels (*P*<0.05). This indicates that both *WRKY18* and *WRKY40* functions are required, but that *WRKY40* over-expression alone is sufficient to partially restore the colonization level of the WT plants.STRING analysis (version 9.0, [33]) was applied to generate a correlation network between *WRKY18*, *WRKY40* and *WRKY60* and other genes, based on co-expression, physical and functional interactions and public based knowledge (Figure 4). Seven out of twenty five genes of the network showed increased expression in the microarray analysis. This is a significant enrichment of up-regulated genes in the network compared to the total number of up-regulated genes represented on the microarray chip (Fisher exact test, p = 2.207E-8). Moreover, by carefully monitoring the expression of all genes in the network using qPCR, we conclude that 12 genes show significant increased expression (p<0.05) 24 hpi (Figure 4). A subset of these 12 genes show also increased expression in time points 9 and 48 hpi (Figure 1A and 1B).

### WRKY18 and WRKY40 positively regulate JA signaling and negatively regulate the expression of *FMO1*, *PAD3* and *CYP71A13* defense genes in response to *Trichoderma*

**Figure 5:**
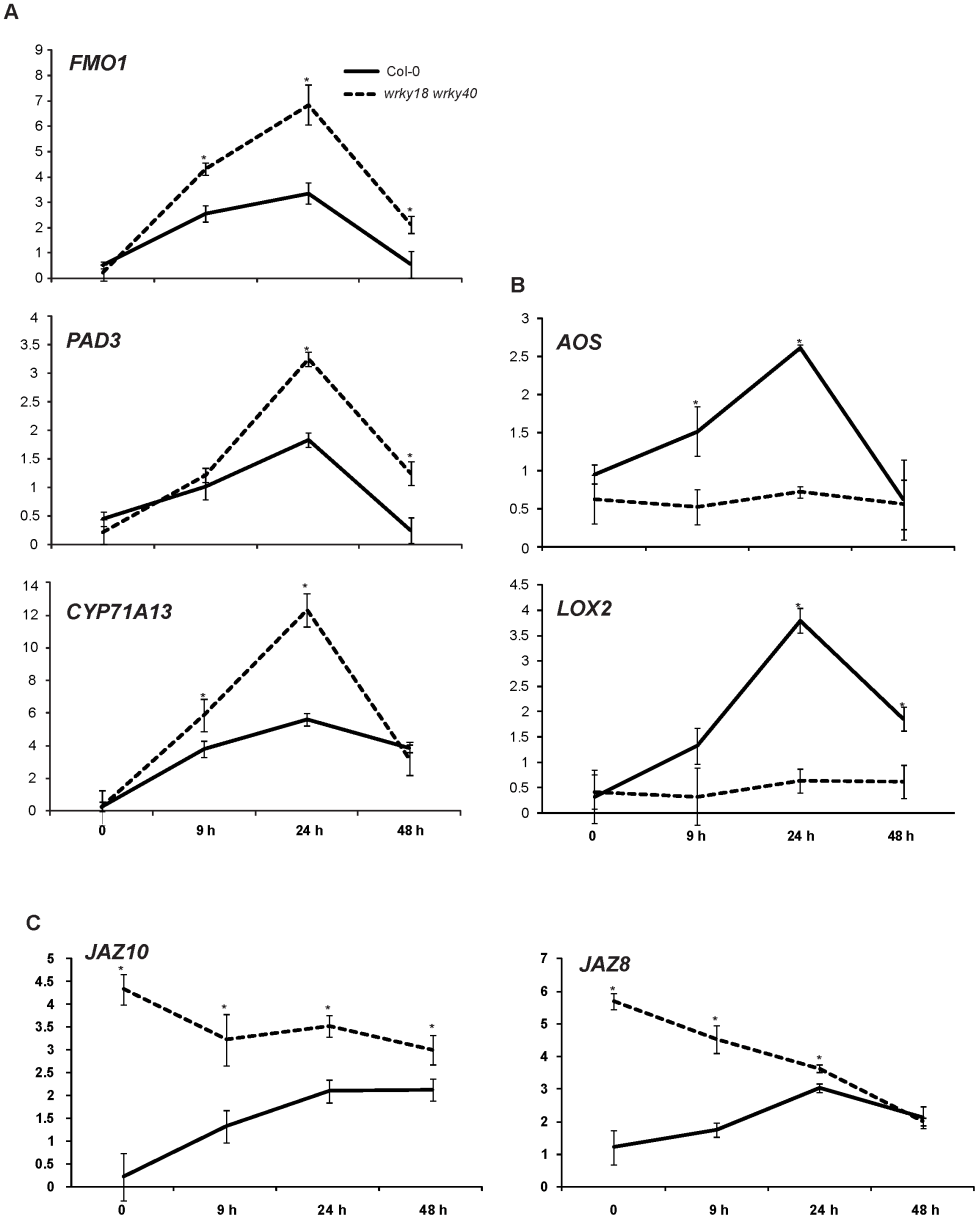
Expression of *Arabidopsis* genes after colonization by *T. asperelloides*. The expression of several genes as determined by qPCR in WT (solid lines) and *wrky18/wrky40* (dashed lines) plants at 9, 24 and 48 h hpi. Gene expression level was calculated with respect to control at each time point. (A) *Fmo1* and *CYP71A13* and *PAD3* (encoding camalexin biosynthesis genes) (B) *AOS* and *LOX2* (encoding JA-biosynthesis genes) (C) *JAZ8* and *JAZ10* (encoding negative regulators of JA signaling). Each time point represents the fold expression average of three independent biological repetitions, and is relative to control collected in each of the specified time points. * significant different (*P*<0.05; *t* test).

To gain better understanding of the role of WRKY18 and WRKY40 in *T. asperelloides* T203 plant root interaction, the expression of *FMO1, PAD3 CYP71A13, AOS, LOX2*, *JAZ8* and *JAZ10* which we found to be up-regulated in *Arabidopsis* WT during root colonization (Figure 1A; File S1), was monitored in the *wrky18/wrky40* double knockout lines inoculated with *Trichoderma*. For *FMO1*, *PAD3*, and *CYP71A13*, we detected higher transcript levels in *wrky18*/*wrky40* than in WT plants (Figure 5A) suggesting that these genes, encoding a SAR regulatory gene and two key genes in camalexin synthesis are under negative control by WRKY18 and WRKY40 in WT plants. WT plants showed 3.8 and 2.8-fold elevated levels of *LOX2* and *AOS* transcripts, respectively at 24 hpi. In contrast, *Trichoderma*-induced expression of both genes was completely absent in *wrky18*/*wrky40* (Figure 5B). These findings suggest that *wrky18*/*wrky40* plants fail to induce the JA pathway. Transcript levels of the *JAZ8* and *JAZ10* genes were elevated in unchallenged *wrky18*/*wrky40* plants (Figure 5C) 9 hours post *Trichoderma* colonization, and their expression declined significantly over the colonization period. In the case of *JAZ8*, transcript reached similar levels to those in WT plants at 48 hpi (Figure 5C). These results may indicate that the failure to induce the JA pathway in *wrky18*/*wrky40* plants may be due to elevated levels of JAZ repressors, and therefore we can assume that WRKY18 and WRKY40 regulate expression of JAZ genes during root colonization.

### *Fmo-1* mutant knockout line shows increased root colonization by *T. asperelloides*

Our findings indicate that upon colonization of the root by *Trichoderma*, WRKY18 and WRKY40 transcription factors regulate the expression of the flavin-dependent monooxygenase1 *FMO1* gene (Figure 5A). To investigate whether *FMO1* has a protective role during root colonization, we evaluated *Trichoderma* root penetration in *FMO1* knockout line (*fmo1*) in comparison to WT Col-0 plants. *Trichoderma* mycelia were recovered from root tissues 24 and 48 hpi and at both time points an increased root colonization was detected in the *fmo1* mutant (Figure 3B). These results, together with data shown previously (Figure 1A, 4 and 5A; File S1), support a model where activation of *FMO1* upon *Trichoderma* root colonization is in part negatively regulated by the WRKY18 and WRKY40 to allow a moderate level of colonization.

### *Trichoderma* root colonization induces changes in the level of indole glucosinolate metabolites

We found that the expression of the *MYB51* gene is induced 9, 24 and 48 hours after the application of *Trichoderma* (Figure 1B) [36]. showed that indole glucosinolates (IGS) biosynthesis is regulated by MYB51, downstream ethylene signaling pathway, and demonstrated the role of 4-methoxy-indol-3-ylmethylglucosinolate (4-methoxy-I3G) in the *Arabidopsis* immune response.

The influence of *Trichoderma* on the level of IGS in *Arabidopsis* roots, 24 hours after colonization, was therefore tested by targeted LC-IT/ESIMS analysis. A significant increase in the level of 4-methoxy-I3G and methoxy-3-indolyl-methylglucosinolate 1MI3MG-1 and decrease in the level of Indolyl-methyl glucosinolate (I3M) was observed (*P*<0.001; Figure S2).

### *Trichoderma* root colonization increases the expression of antioxidant enzymes and the ascorbic acid pool during salt stress

**Figure 6:**
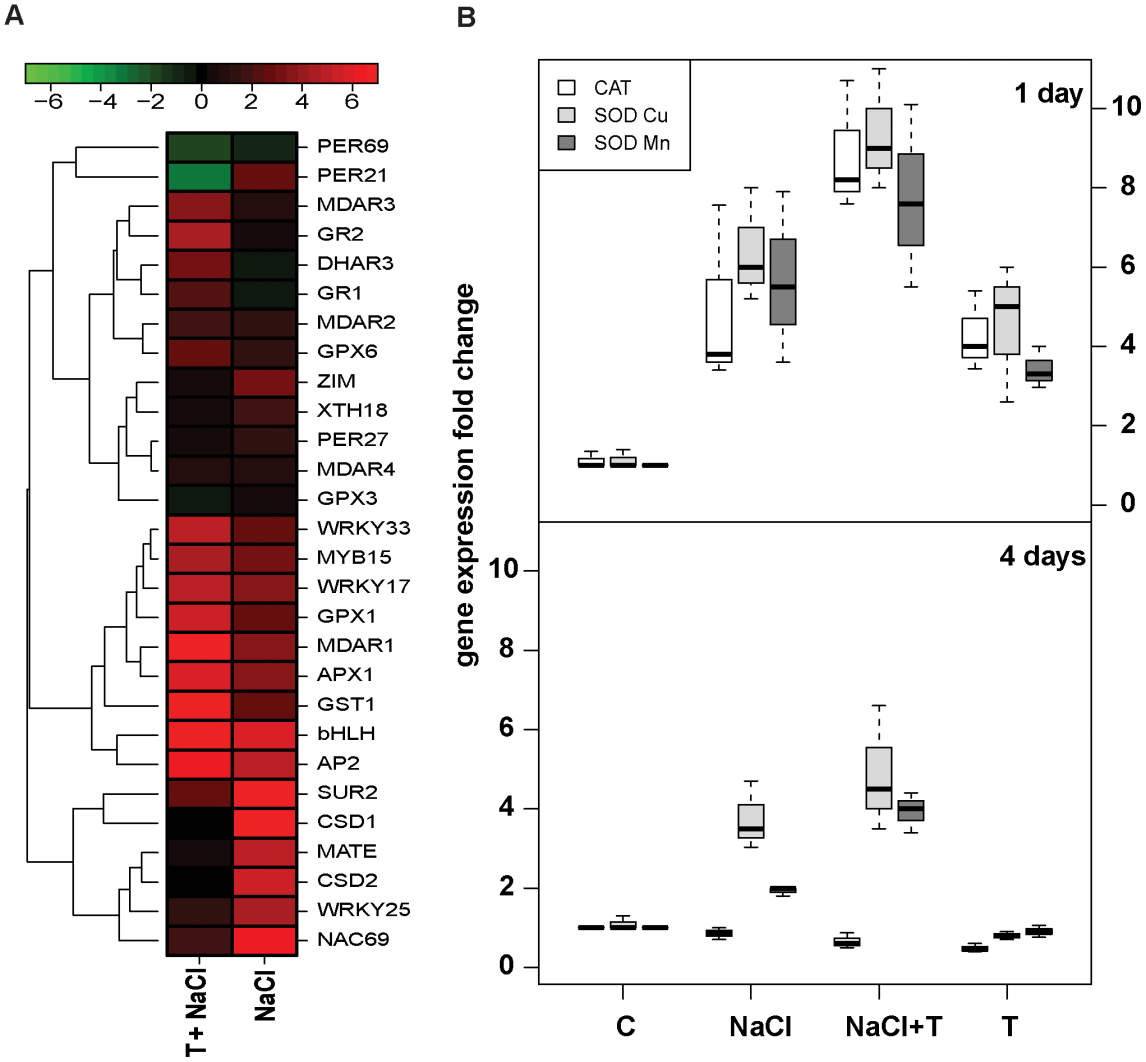
qRT-PCR expression analysis of antioxidant enzymes in cucumber seedlings and roots of four weeks old *Arabidopsis* under salt stress. (A) RNA was extracted from untreated *Arabidopsis* roots and from roots collected from plants exposed to 100 mM NaCl (NaCl), and from roots colonized by T203 for 48 hours prior exposure to 100 mM NaCl (T+NaCl). Hierarchical clustering by Euclidean distance method average linkage is shown. The color of the each cell indicates fold-change relative to control as follows: Red: significant up regulation (*P*<0.05; *t* test), green: significant down regulation (*P*<0.05; *t* test). Black: not statistically significant difference from the control,. (B) RNA was extracted from untreated cucumber seedlings (Control), from seedlings exposed to 100 mM NaCl (+NaCl), from seedlings colonized by T203 (+T) and from seedlings colonized by T203 for 48 hours prior exposure to 100 mM NaCl (+T+NaCl). RNA was extracted 1 day and 4 days after salt addition. Fold expression (for A and B) was calculated from average of three independent biological repetitions.

An increased expression of genes that function in general tolerance to abiotic stresses (File S1) was pointed out by the results of the microarray analysis. Among them, genes that take part in salt tolerance and osmoprotection processes (File S3). To further test *Trichoderma*-induced plant response to salt stress, the expression of 28 *Arabidospis* genes previously identified as NaCl responsive genes [37] and related to osmoprotection, or to general response to oxidative stress, was monitored by qPCR in control plants and plants subjected to salt stress (100 mM NaCl) with or without *Trichoderma* pre-treatment 48 hours before salt stress imposition (Figure 6A). Worthy of note was the induced expression of *MDAR*, *APX1* and *GST* indicating a general activation of the plant antioxidant machinery by *Trichoderma*. Activation of the antioxidant defense by *Trichoderma* was found also in cucumber (*Cucumis sativus*) seedlings subjected to salt stress (100 mM NaCl) with or without *Trichoderma* pre-treatment. The expression of the *cat*, *sod(Mn)* and *sod(Cu)* genes was analyzed by qRT-PCR, one and four days after salt addition (Figure 6B). After 24 hours the expression of all tested genes was increased (5–8 fold) by the salt addition and also by *Trichoderma* root colonization. Enhanced expression of the genes could be detected in seedlings that were infected with *Trichoderma* 48 hours prior the salt addition (+T+NaCl). After four days (Figure 6B) the *cat* gene expression is decreased in all treatments, while the expression of the *sod (Mn)* and *sod(Cu)* genes remains elevated in the plants under salt stress and even higher in the *Trichoderma* pretreated plants. In the absence of stress imposition, higher ascorbate levels in its reduced form were measured in *Trichoderma* treated cucumber plants, whereas levels of dehydroascorbate were reduced giving a higher AA/DHA ratio (Table 2). Accordingly, the relative *MDAR* expression level is increased by 15 fold in cucumber plants treated with *Trichoderma* for 48 hours, as measured by qPCR. Upon saline stress imposition in the presence of *Trichoderma* the levels of reduced and oxidized forms of ascorbic acid were comparable to those of control plants, confirming the protective effect of *T. asperelloides* T203.

**Table 2:** Measurement of reduced, oxidized (dehydroascorbate) and total ascorbate content in cucumber seedlings and the ASC/DHA ration

Treatment:	Control	+NaCl	+T203	+T203+NaCl
AA (reduced)	317 (±3.5)^A^	273.7 (±9)^B^	384 (±8)^C^	320 (±5)^A^
DHA(oxidized)	21.3 (±3.2)^A^	24.3 (±3)^B^	15.3 (±3.5)c	22 (±3.5)^AB^
AA/DHA	15 (±2)^A^	11.4 (±1.8)^B^	25.5 (±4.4)^C^	14.6 (±1.4)^A^

Cotyledons were sampled from seven days old seedlings grown in soil with or without *Trichoderma* (Control and +T203) with or without 75 mM NaCl (+NaCl and +T203 +NaCl). Quantification of the reduced (AA) and oxidized (DHA) forms of ascorbate in plants was done as describe in material and method. The ratio between the reduced and oxidized form of ascorbic acid is shown (AA/DHA). Results are average of three independent pools of plant material ± standard deviation. Statistically significant differences between treatments (*P*<0.05) are marked with distinct letters (A,B,C,D) for AA, DHA and AA/DHA.

### *Trichoderma* wild-type but not ACC-deaminase silenced mutants can improve cucumber and *Arabidopsis* seed germination under salt stress conditions

**Figure 7:**
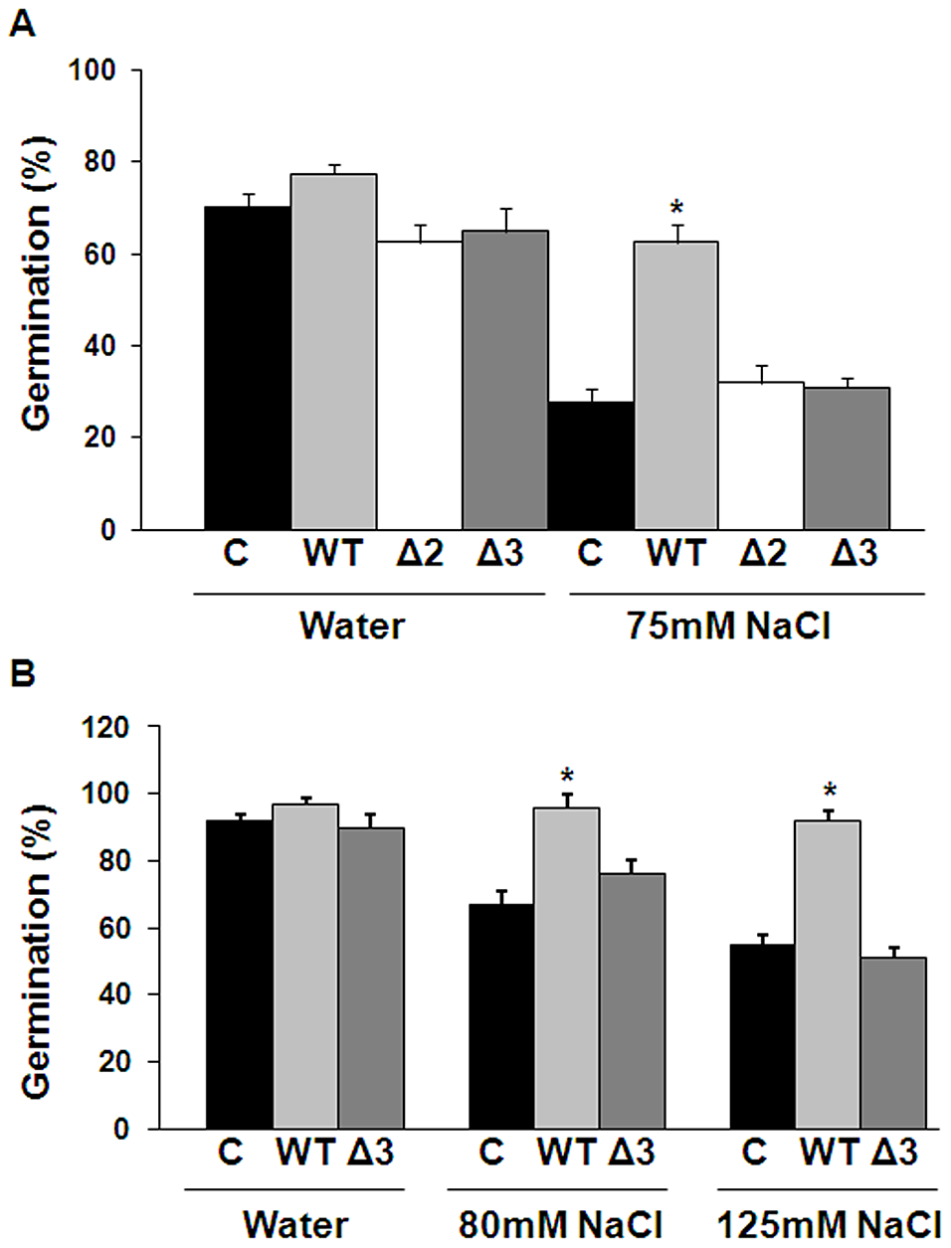
Germination (%) of cucumber and *Arabidopsis* seedlings under salt stress conditions. (A) Cucumber seeds were planted in untreated soil (Control) or in pots with soil mixed with a spore suspension (10^6^spores/g soil) of T203–WT, or ACC-deaminase silenced mutants ΔACC#2 and ΔACC#3. The pots were watered with either tap water or a solution of 75 mM NaCl. Germinating seedlings were counted 7–10 days after planting. (B) *Arabidopsis* seeds were planted as described for the cucumber seeds and watered with either tap water, 80 mM or 125 mM NaCl solution.

The ability of *Trichoderma* to ameliorate seed germination under saline stress conditions was assessed in soil. In absence of saline stress cucumber (Figure 7A) or *Arabidopsis* (Figure 7B), seeds planted in soil treated with *Trichoderma* T203 (WT) or with ACC-deaminase mutant spores showed similar germination rates to those of the control seeds treatment. However, when cucumber (Figure 7A) or *Arabidopsis* (Figure 7B) seeds were treated with saline solutions, the germination rate was significantly higher in *Trichoderma* WT treated soil than in untreated (control) or ACC-deaminase mutant treated soil. These results indicate that the afforded tolerance to the salt application is probably linked to a lower level of deleterious ethylene evolving under stress conditions.

## Discussion

 Relatively little is known about the host mechanisms that connect the perception of *Trichoderma* root colonization to the downstream signaling pathways leading to activation of defense and developmental responses. It is assumed that microbe- associated molecular pattern (MAMP) recognition triggers the activation of a signaling cascade that activates a variety of defense responses including callose deposition, programmed cell death, production and accumulation of antimicrobial reactive oxygen species, and induction of phytoalexins and other secondary metabolites [5].

In the current study, microarray analysis of *Arabidopsis* roots colonized by *Trichoderma*, coupled with qPCR analysis, allowed identification of genes involved in activation of the first stage of root colonization by the fungus, plant growth responses, biotic resistance and abiotic tolerance induced by this beneficial fungus.

*Trichoderma* effects on plant growth promotion and root architecture are well known [8], [38]. *Trichoderma* enhanced biomass production and lateral root growth promotion were shown to be an auxin-dependent mechanism in *Arabidopsis* [39]. In the present study we found that MYB77, which is involved in auxin response and later root formation [40], and ASA1, that functions in jasmonate mediated regulation of auxin biosynthesis and transport during lateral root formation [41], are up-regulated during *Trichoderma* root colonization (Figure 1A and 1B, Figure 2). Interestingly, microarray results indicate activation of other genes related to root and plant development. Among them are *ATPSK2*, involved in cell proliferation and organ morphogenesis [42], [43], and the transcription factor *ANAC081*, known to be linked to increased leaf size and biomass traits [44].

Genes with known role in plant defense responses are up-regulated during *Trichoderma* colonization (File S1), for example, *PR-2* and *PR-5*. Interestingly, prominently four genes (*PDF1*, *PDF1.2*, *PDF.1.2c*, *PDF1.3*) out of thirteen members of the *Arabidopsis* defensin gene family, that inhibit the growth of a broad range of fungi, [45], are up-regulated. These findings are comparable to similar results obtained by [46].

Increase in transcript abundance of *MYB72* (Figure 1B) is in agreement with the results obtained by [47] which demonstrated the role of MYB72 in the early signaling steps of *Trichoderma* mediated ISR.

The ethylene dependent MYB51 which is involved in the transcriptional activation of indole glucosinolate (IGS) biosynthetic genes during plant defense responses [36] is transiently up-regulated by *Trichoderma* with highest expression at 24 hours after the application of *Trichoderma* to the roots. An increase in the expression of *CYP79B3*, that take part in the conversion of tryptophan to indole-3-acetaldoxime a precursor of indole glucosinolates and the anti-microbial molecule camalexin, was observed too (Fig. 1A).

Because CYP79s catalyze a key step in the biosynthesis of glucosinolates, plant products that function in the defense toward herbivores and pathogens, alteration of the expression of these genes often has dramatic effects on the profile of glucosinolates. To further exploit the induction in the expression of *MYB51* and CYP79s genes, the content of three key IGS metabolites was measured in *Trichoerma* treated and control *Arabidopsis* roots. Our results show a significant increase in the level of 4-methoxy-I3G and 1MI3MG-1 and decrease in the level of their precursor I3M (Figure S2). Similarly [36], showed that Flg22 elicited biosynthesis of 4-methoxy-I3G, associated to MYB51 activity.

Interestingly, two other CYP genes beside *CYP79B3*, *CYP71B15* and *CYP71A13*, that function in camalexin biosynthesis [48], are significantly affected by *Trichoderma* root colonization (Figure 1B).

Among the 28 transcripts down-regulated 24 hours after *Trichoderma* inoculation, four of them are plant cytochrome P450 monooxygenases (*CYP712A2*, *CYP712A1*, *CYP93D1* and *CYP76G1*). These genes mediate synthesis and metabolism of many physiologically important primary and secondary compounds that are related to plant defense against a range of pathogenic microbes and insects [49]. Their down-regulation could indicate a strategy for repressing local defense responses to allow successful colonization as recently reported for *P. indica* [22], [50]. This strategy is also supported by the induced expressions of the transcription factor ANAC081, which has been shown to be a repressor of the expression of genes that encode pathogenesis-related proteins, and the over-expression of this TF causes susceptibility to the soil-borne fungal pathogen *Fusarium oxysporum* [44].

Transcriptional activation upon pathogen attack is a common feature of most of the group III *WRKY* genes. Furthermore, most of these genes are expressed during both non-host and *R* gene–dependent resistance [51]. Three WRKY group III transcription factors (WRKY41, WRKY53, and WRKY55) are up-regulated during the first 24 hours of *Trichoderma* root colonization, but their expression goes down together with expression of other defense related transcripts, again supporting the idea that *Trichoderma* somehow can temporarily repress local defense plant immune response [20].

*Trichoderma* root colonization triggers also a rapid increase in transcription factors expression like *WRKY18, WRKY40*, *WRKY60* and *WRKY33*, which activate JA-pathway responses and represses SA signaling. *WRKY18*, *WRKY40* and *WRKY60* are pathogen-induced and encode three structurally related WRKY proteins which exert a positive role in JA-mediated defense [28], [30]. *Trichoderma* regulate the expressions of the *WRKY18* and *WRKY40* TFs to allow root colonization, in a similar way to the biotrophic fungus *Golovinomyces orontii* and the hemibiotrophic bacterium *Pseudomonas syringe* and in contrast to necrotrophic fungus *Botrytis cinerea* [28], [29]. Hence, beneficial microorganism and plant pathogens share common molecular mechanism to cope with the plant immune system.

Interestingly, our findings resemble the interaction observed during the early stages of infection of *Arabidopsis* by *G. orontii* [30]. In the course of infection, *G. orontii* manipulate WRKY18 and 40 transcription factor activities to modulate the expression of JAZ repressor genes and defense response genes, such as *FMO1* and *CYP71A13* to its advantage. Recently [20], in microarray analysis of aerial parts of *Arabidopsis* plants 24 hours after application of *T. harzianum* T34 showed reduced expression of the genes *FMO1* and *CYP71A13*. Although T203 colonization induces the expression of *FMO1* and *CYP71A13* in *Arabidopsis* WT (Col-0) plants (Figure 1A, Figure 5A, File S1), monitoring those genes expression in *wrky18/wrky40* double knockout line showed a significant higher expression upon colonization by T203 (Figure 5A). Hence, increased T203 colonization of the *fmo1* knockout line (Figure 3B) provides further evidence for a model were *Trichoderma* spp. fine-tune the expression of defense genes such as *FMO1* and *CYP71A13* to allow colonization.

*FMO1*, the flavin-dependent monooxygenase, was reported to be essential for the initiation of systemic resistance in *Arabidopsis* [52], [53], although its deletion did not affect local defense responses at the site of pathogen attack. It was suggested that a metabolite generated by FMO1 might be necessary during the early phase of SAR establishment and that FMO1 contributes to a signal amplification loop required to potentiate SAR responses in systemic tissues. The involvement of FMO1 in *Trichoderma* mediated ISR cannot be excluded too. T-DNA insertion into the *FMO1* gene resulted in enhanced susceptibility to virulent *Pseudomonas syringae* and *Hyaloperonospora parasitica* [54]. In our study, the same T-DNA insertion line revealed increased colonization by *T. asperelloides* (Figure 3B), thus showing FMO1 function in defense response also in roots, and how its function is essential to balance the level of root colonization by *T. asperelloides*.

MYB51, MYB15 and WRKY33 are also key elements in the plant response to abiotic stresses. WRKY33 over-expression was found sufficient to increase *Arabidopsis* tolerance to NaCl and to regulate transcription of several downstream genes involved in the response to stress [55]. This and other molecular changes induced in the plant by *Trichoderma* root colonization are in good correlation with the protective effect induced in plants by these beneficial fungi towards a plethora of environmental stresses. Plants contain a series of enzymatic antioxidants such as superoxide dismutase (SOD), ascorbate peroxidase (APX; EC 1.11.1.11), and catalase. These antioxidants function properly to interrupt the cascades of uncontrolled oxidation in some organelles and to scavenge the toxic ROS produced under environmental stresses.

We could show that in cucumber the expression of *cat* and *sod* genes is affected by *Trichoderma* colonization of the roots resulting in long lasting up-regulation upon salt stress imposition (Figure 6B). Besides these enzymatic mechanisms, low molecular mass antioxidants are efficient radical scavengers and may play a role in oxidative stress response of higher plants. Ascorbate is a major antioxidant that is involved in the ascorbate-glutathione cycle. Higher levels of ascorbate in its reduced form were found in *Trichoderma* treated plants (Table 2), similarly to data reported for plant root inoculation by *P. indica* [12]. Monodehydroascorbate reductase (MDAR) is the enzymatic component involved in the regeneration of reduced ascorbate. MDAR was shown to be crucial for the mutualistic interaction between *Arabidopsis* and *P. indica* [11]. Interestingly, this gene is also highly induced in cucumber and *Arabidopsis* during root colonization by *Trichoderma* (Figure 6A, File S1). In *Arabidopsis*, *Trichoderma* pre-treatment prior salt stress imposition also stimulates expression of various transcripts involved in osmoregulation and general oxidative stresses (Figure 6A). The mechanism proposed in this study according which *T. asperelloides* T203 can induce plant tolerance to salt stress has been proposed recently, also by [8] in relation to the tolerance afforded to tomato seedlings to water deficit by pre-treatment with *T. harzianum* T22. In consequence, the activation of the antioxidant machinery in order to recycle oxidized ascorbate appears to be a general mechanism activated by different *Trichoderma* strains (*T. asperelloides* and *T. harzianum*) in different plant species (*Arabidopsis*, cucumber and tomato) to enhance tolerance to a range of abiotic stresses.

Beside the activation of the antioxidant machinery, the microarray results show also activation of other genes, which have protective role in *Arabidopsis* against salinity and osmotic stress (File S3). For example, we detected up regulation of two members of the aquaporin gene family (*AT2G34390* and *AT2G29870*). Heterologous over-expression of rice and wheat aquaporin genes in *Arabidopsis* resulted in increase tolerance to salinity and dehydration [56], [57].

Biosynthesis of ethylene in plants under salinity stress is well established. Higher ethylene concentration inhibits root growth and ultimately affects the overall plant growth. Many studies have shown that ethylene level in plants is regulated by a key enzyme 1-aminocyclopropane-1-carboxylicacid (ACC)-deaminase. This enzyme present in plant growth-promoting bacteria (PGPR) and other microorganisms [58], lowers the ethylene level by metabolizing its precursor ACC into 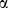-ketobutyrate and ammonia (NH_3_).

Inoculation of plants under salinity stress with PGPR having ACC-deaminase activity mitigates the inhibitory effects of salinity on root growth by lowering the ethylene concentration in the plant. This in turn results in prolific root growth, which is beneficial for the uptake of nutrients and maintenance of growth under stressful environment [59].

In a previous work we presented data based on *Trichoderma* silenced mutants suggesting a central role for ACC deaminase (ACCD) activity in the plant growth promotion effect by *T. asperelloides* T203 [14]. Here we show that the same mutants are not able to afford tolerance, both to cucumber and *Arabidopsis*, during germination under salt stress imposition (Figure 6). This suggest that *Trichoderma* fungi, similarly to PGPR bacteria, can ameliorate plant growth under abiotic stressful conditions by lowering deleterious elevated ethylene levels accompanied by an elevated antioxidative capacity.

As mentioned above, *Trichoderma* stimulate biomass production and lateral root growth promotion by production of auxin [39]. This is supported by induction upon *Trichoderma* colonization of genes such as *ASA1* and *MYB77* (Figure 1A and 1B). In the root, ethylene and auxin can reciprocally regulate each other's biosyntheses [60], [61] proposed that *Trichoderma* IAA contributes to exogenous auxin-stimulated ethylene biosynthesis via ACC synthase. In this model, *Trichoderma* ACCD activity reduces the availability of ACC necessary for ethylene biosynthesis and the reductions in ethylene promote plant growth via gibberellins (GA) signaling by increasing the degradation of DELLA proteins that are repressors of gibberellin (GA) signaling. Moreover, gibberellins may control the onset of JA- and SA-dependent defense responses of the plant through the regulation of DELLA protein degradation [60]. It thus appears that defense occurs at the expense of growth. Supporting this view, recent studies have uncovered new roles for both JAZ and DELLA proteins in the regulation of JA-GA crosstalk as well as the conflicting association between defense and growth. DELLAs' positive effect on JA signaling seems to be exerted at the level of JAZ repressors as DELLAs interact with JAZ proteins and hinder their ability to repress MYC2 [62], [63]. Since MYC2 has not a significant change in our present work (Figure 4) it seems that during root colonization, growth is promoted through GA-mediated degradation of DELLAs while defense is repressed through JAZs repressing MYCs. This tips the balance towards growth, while allowing root colonization by *Trichoderma*. 

## Materials and Methods

### Plant material and fungal strain

*Trichoderma asperelloides* T203 [64] and *Trichoderma asperelloides* 1-Aminocyclopropane-1-carboxylate (ACC)-deaminase silenced mutants (ΔACC#2; ΔACC#3) which were created in a previous study [14], were routinely propagated and sporulated on Potato Dextrose Agar (PDA) plates. *Arabidopsis thaliana* Col-0 ecotype plants were used throughout this work. *WRKY18 and WRKY40* double knock-out (*wrky18*/*wrky40*) and the WRKY40-over-expressing lines are kind gifts from Prof. Somssich [30]. The *FMO1* knock-out (*fmo1*) line is a precious gift from Prof. Schlaich [54]. Cucumber seeds (*Cucumis sativus* L. cv. Kfir) were purchased from Gedera Seeds Co. (Israel).

### Fungal and plant growth conditions

For microarray and gene expression analysis by qPCR, *Arabidopsis* Col-0, *wrky18*/*wrky40*, WRKY40-over-expressingline and the *fmo1* mutant plants were grown 25 days under a long day regime (16 hours light), on rock-wool placed over a 5 L tank supplemented with *Arabidopsis* hydroponic solution [19]. *Trichoderma* inoculum was added to the root system, resulting in a concentration of 10^5^ germinated spores mL^−1^. Roots were collected at 9, 24 and 48 hpi. For salt stress imposition in *Arabidopsis*, NaCl was added to the growth medium at a final concentration of 100 mM, two days after *Trichoderma* root inoculation and roots were collected after two more days. Cucumber seedlings were grown in hydroponics boxes as described in [4]. For cucumber salt stress imposition NaCl was added at a final concentration of 100 mM two days after *Trichoderma* root inoculation.

For greenhouse experiments, cucumber or *Arabidopsis* seeds were planted in soil in 250 ml boxes. *Trichoderma* strains were mixed with the soil at a final concentration of 10^6^spores/g soil. Seeds were watered with NaCl solutions at concentrations described in each experiment, or water (control) starting from seeding. Seedlings germination was evaluated 7 days from planting. Each treatment included 4 boxes with 10 cucumber seeds or 20 *Arabidopsis* seeds. The experiment was repeated three independent times.

### RNA extraction and microarray analysis

For microarrays analysis total RNA was purified using the RNeasy Mini Kit (Qiagen) from *Arabidopsis* roots pooled from 60 plants (24 hpi and from non-inoculated control plants) from two independent experiments. DNaseI digestion was performed with the Turbo DNA-free Kit (Ambion). cDNA was prepared and hybridized to Agilent *Arabidopsis* (V4) Gene Expression Microarrays (4×44K) in the Microarrays Unit of the Weizmann Institute of Science (Rehovot, Israel), according to manufacturer instructions. The microarray data have been deposited in the Gene Expression Omnibus (GEO) database, www.ncbi.nlm.nih.gov/geo (Accession no. GSE42113). Statistical analysis, normalization and fold-change values of microarray data were performed using the ROBIN software [65]. The MapMan software was used in order to create MapMan overview diagrams of the microarray data [34]. Twenty-two genes (list of primers in File S5) were used to validate the microarray analysis by qPCR as described in the following section.

### Expression profiling by qPCR

Total RNA was prepared as described in the previous section from roots of 35 plants. Four micrograms of total RNA were used as template for first-strand cDNA synthesis with RevertAid cDNA Synthesis Kit (Thermo Fisher Scientific). cDNA (20 ng) was used for qPCR with Power SYBR Green reagent performed on a ABI PRISM 7900HT sequence detection system (Applied Biosystems). Data were analyzed with the 7900 V2.0.3 evaluation software (Applied Biosystems).

All genes and primers used in this paper are listed in file S4. *Arabidopsis* primers sequences were designed using the QuantPrime online tool (www.quantprime.de) and available through the Mueller-Roeber Lab webpage. The 137 genes of the *Arabidopsis* expression platform have been selected by the criteria of being experimentally proven to be involved in defense response processes against biotic and abiotic stress. Cucumber primers were designed using Primer Express (Applied Biosystems). The fold change in the target genes was normalized to ACTIN2 and GADPH reference genes for *Arabidopsis* and the 18S reference gene for cucumber. Gene fold expression relative to control plants was determined using the ΔΔCT as described in [66]. Three biological experiments (with two independent replicates, obtain from different roots pool, for each experiment) were performed for each treatment.

### Quantification of *Trichoderma* root colonization by qPCR and root colonization assay

A time-course experiment was performed with inoculated *Arabidopsis* roots detached from 35 plants from each hydroponic growth container at 24, 48, 72, 96 and 120 hpi extensively washed in water. Plant and Fungal growth conditions as described above. After sterilization in 1% (v/v) NaOCL for 1 min, the roots were washed with sterile distilled water, and total DNA extraction was performed according to [67]. *In planta* quantification of *T. asperelloides* was followed by amplification of 200-bp and 201-bp using gene specific primers for the *Trichoderma* genes *TasSwo* [68] and *β*-tubulin, respectively. Primers sequences can be found in file S4. *Arabidopsis* ACTIN2 and GADPH genes) were used as control references for quantitative analysis. qPCR was carried out as mentioned above. Each sample was examined in triplicates using a relative quantification analysis by the standard curve technique as described by [35], [69]. Root colonization assays were performed according to [70]. Briefly, roots were detached 12 h post-inoculation and extensively washed in water. After sterilization in 1% NaOCl for 2 min, the roots were washed with sterile distilled water, weighed, and homogenized using an ULTRA-TURRAX apparatus (Janke & Kunkel) in 20 mL of water for 1 min. Serial dilutions were plated for colony forming unit counts on *Trichoderma* selective medium [71] at 28°C.

### LC-MS analysis of indole glucosinolates metabolites

Secondary metabolite analysis by LC-MS was performed as described by [72]. All data were processed using Xcalibur 2.1 software (Thermo Fisher Scientific, Waltham, USA). Indole glucosinolates identification and annotation were performed using comparison with our previous publications [73], [74].

### Ascorbic acid determination

Measurement of reduced, oxidized and total ascorbate content in plants was done according [75] using the α-α′-bipyridyl method. Cotyledons were sampled from seedlings grown in soil with or without *Trichoderma* (C and +T) with or without salt stress application (+NaCl and +NaCl+T). Plant material was pooled from three different plants, ground, and 200 mg were taken in the assay.

### Statistical analysis

For seedlings germination assay and ascorbic acid determination JMP7 software (SAS Institute Inc.) was used for statistical analyses. Data were analyzed using one-way ANOVA and mean comparisons were made using the Tukey–Kramer honestly significant difference multiple range test at (*p*<0.05). For qPCR analysis genes expression data were analyzed with one-side, unpaired Welch's *t*-test (*p*<0.05) in Excel. For microarrays analysis Welch's *t*-test was applied to identify differentially expressed genes. Gene lists were created by filtering the genes based on fold change and signal above background in at least one microarray. Up-regulated genes were defined as those having a greater than or at least two-fold linear intensity ratio. For indole glucosinolates determination data were analyzed with one-side, unpaired Welch's *t*-test (*p*<0.001) in Excel.

## Supporting Information

Figure S1 MapMan screenshot showing the effects of *Trichoderma* root inoculation on the root transcriptome. The MapMan software was queried with the list of differentially regulated genes 24 hours after the application of *Trichoderma*. Blue shades indicate induction; Red shades indicate repression of gene expression.
(PNG)

Figure S2 Targeted LC-IT/ESIMS based quantification of indole glucosinolates in *Arabidopsis* roots 24 hours after colonization by *T. asperelloides*. Abbreviation: 4MI3MG, 4-methoxy-indol-3-ylmethylglucosinolate; 1MI3MG, 1-methoxy-3-indolyl-methyl glucosinolate; I3M, Indolyl-methyl glucosinolate. Each of the glucosinolate shows a significant difference (P<0.001) between the control and *T. asperelloides* treatment. Results are averages (± standard deviation) of six replicates from two independent biological repetitions. Each repetition was a pool of 35 plants.
(TIF)

File S1 List of genes and fold changes that are significantly differentially expressed in *Arabidopsis* roots 24 hours after application of *Trichoderma* as monitored by microarray analysis.
(XLS)

File S2 Complete overview of statistically significant enriched biological processes.
(XLS)

File S3 A summary of the genes up-regulated in the microarray experiments with known role in plant hormone biosynthesis process, responses to hormone stimulation, salt tolerance and osmoprotection processes.
(XLS)

File S4 Sequences of all primers used in qPCR analyses.
(XLS)

File S5 qPCR verification of microarray data.
(XLS)
