# Correction: *Trichoderma*-Plant Root Colonization: Escaping Early Plant Defense Responses and Activation of the Antioxidant Machinery for Saline Stress Tolerance

**DOI:** 10.1371/annotation/8b818c15-3fe0-4e56-9be2-e44fd1ed3fae

**Published:** 2013-04-30

**Authors:** Yariv Brotman, Udi Landau, Álvaro Cuadros-Inostroza, Tohge Takayuki, Alisdair R. Fernie, Ilan Chet, Ada Viterbo, Lothar Willmitzer

The fourth author's name was written incorrectly, with the first and last name reversed. The correct name is: Takayuki Tohge. The correct citation is: Brotman Y, Landau U, Cuadros-Inostroza Á, Tohge T, Fernie AR, et al. (2013) Trichoderma-Plant Root Colonization: Escaping Early Plant Defense Responses and Activation of the Antioxidant Machinery for Saline Stress Tolerance. PLoS Pathog 9(3): e1003221. doi:10.1371/journal.ppat.1003221

