# Supplementary figures and images for: Trichoderma-Plant Root Colonization: Escaping Early Plant Defense Responses and Activation of the Antioxidant Machinery for Saline Stress Tolerance
Source: PLoS Pathog. 2013 Mar 14;9(3):e1003221. doi: 10.1371/journal.ppat.1003221 (PMC3597500; doi:10.1371/journal.ppat.1003221)

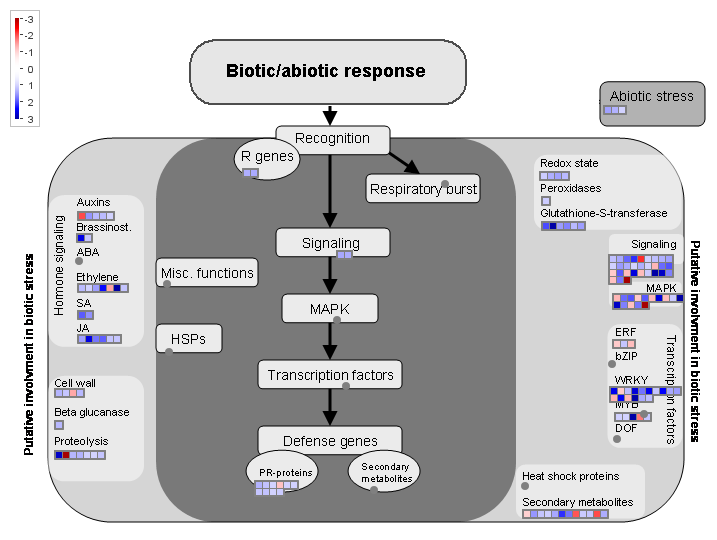

Supplement: Figure S1 — MapMan screenshot showing the effects of Trichoderma root inoculation on the root transcriptome. The MapMan software was queried with the list of differentially regulated genes 24 hours after the application of Trichoderma. Blue shades indicate induction; Red shades indicate repression of gene expression. (PNG) [file ppat.1003221.s001.png]

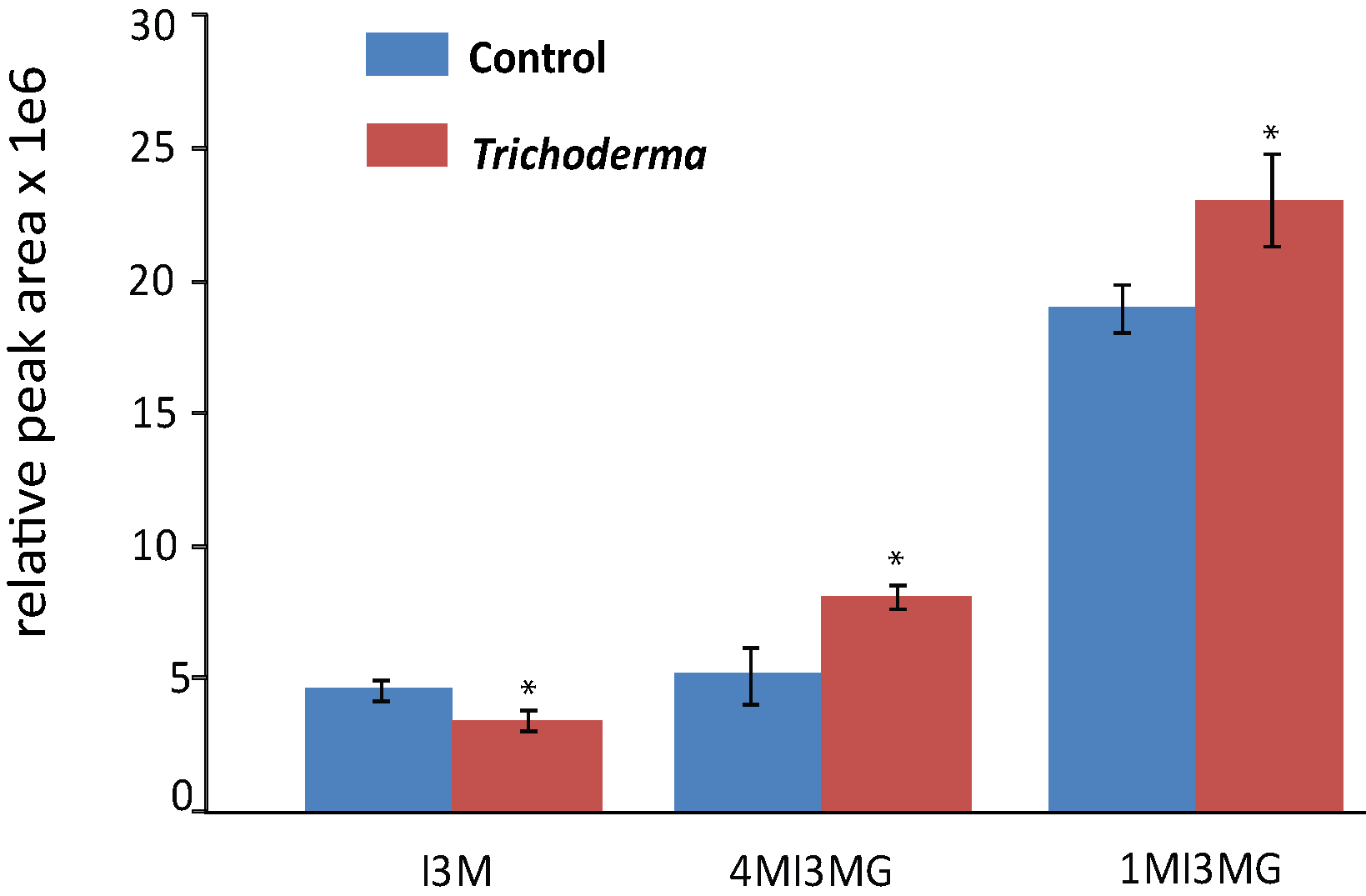

Supplement: Figure S2 — Targeted LC-IT/ESIMS based quantification of indole glucosinolates in Arabidopsis roots 24 hours after colonization by T. asperelloides. Abbreviation: 4MI3MG, 4-methoxy-indol-3-ylmethylglucosinolate; 1MI3MG, 1-methoxy-3-indolyl-methyl glucosinolate; I3M, Indolyl-methyl glucosinolate. Each of the glucosinolate shows a significant difference (P<0.001) between the control and T. asperelloides treatment. Results are averages (± standard deviation) of six replicates from two independent biological repetitions. Each repetition was a pool of 35 plants. (TIF) [file ppat.1003221.s002.tif]
